# Comparative biology and population mixing among local, coastal and offshore Atlantic herring (*Clupea harengus*) in the North Sea, Skagerrak, Kattegat and western Baltic

**DOI:** 10.1371/journal.pone.0187374

**Published:** 2017-10-30

**Authors:** Florian Berg, Aril Slotte, Arne Johannessen, Cecilie Kvamme, Lotte Worsøe Clausen, Richard D. M. Nash

**Affiliations:** 1 Institute of Marine Research (IMR), Nordnes, Bergen, Norway; 2 University of Bergen, Department of Biology, Bergen, Norway; 3 International Council for the Exploration of the Sea, H. C. Andersens Boulevard, Copenhagen, Denmark; Maurice Lamontagne Institute, CANADA

## Abstract

The population structure of Atlantic herring (*Clupea harengus*) from 13 local, coastal and offshore areas of the North Sea, Skagerrak, Kattegat and western Baltic (northeast Atlantic) was studied using biological and environmental data from 1970–2015. The objective was to identify distinct populations by comparing variability in the temporal and spatial phenotypic characteristics and evaluate the potential for mixing of populations in time and space. The populations varied in biological characteristics such as mean vertebral counts (VS), growth and maturity ogives. Generalized additive models indicated temporally stable VS in the North Sea and western Baltic, whereas intra-annual temporal variation of VS occurred in other areas. High variability of VS within a population was not affected by environmental factors such as temperature and salinity. Consequently, seasonal VS variability can be explained by the presence or absence of herring populations as they migrate between areas. The three main populations identified in this paper correspond to the three managed stocks in this area: Norwegian spring spawners (NSS), western Baltic spring spawners (WBSS) and North Sea autumn spawners (NSAS). In addition, several local populations were identified in fjords or lakes along the coast, but our analyses could not detect direct mixing of local populations with the three main populations. Our results highlight the importance of recognizing herring dynamics and understanding the mixing of populations as a challenge for management of herring.

## Introduction

In many coastal and offshore areas, fish originating from different spawning populations mix during non-spawning seasons and can be targeted simultaneously by fisheries. Such mixing can be a challenge for fisheries managers who often prefer to use the term ‘stock’ in the management context instead of population. Throughout this paper, the term ‘stock’ is used in the management context, defined by International Council for the Exploration of the Sea (ICES) [1] as “a part of a fish population (or several populations) usually with a particular migration pattern, specific spawning grounds, and subject to a distinct fishery. In theory, a ‘stock’ comprises all the individuals of fish in an area, which are part of the same reproductive process”. This definition does not necessarily imply that a stock consists only of a single population. Here we use, a ‘population’ as a reproductive ‘unit’ of herring in the evolutionary context [2].

**Fig 1:**
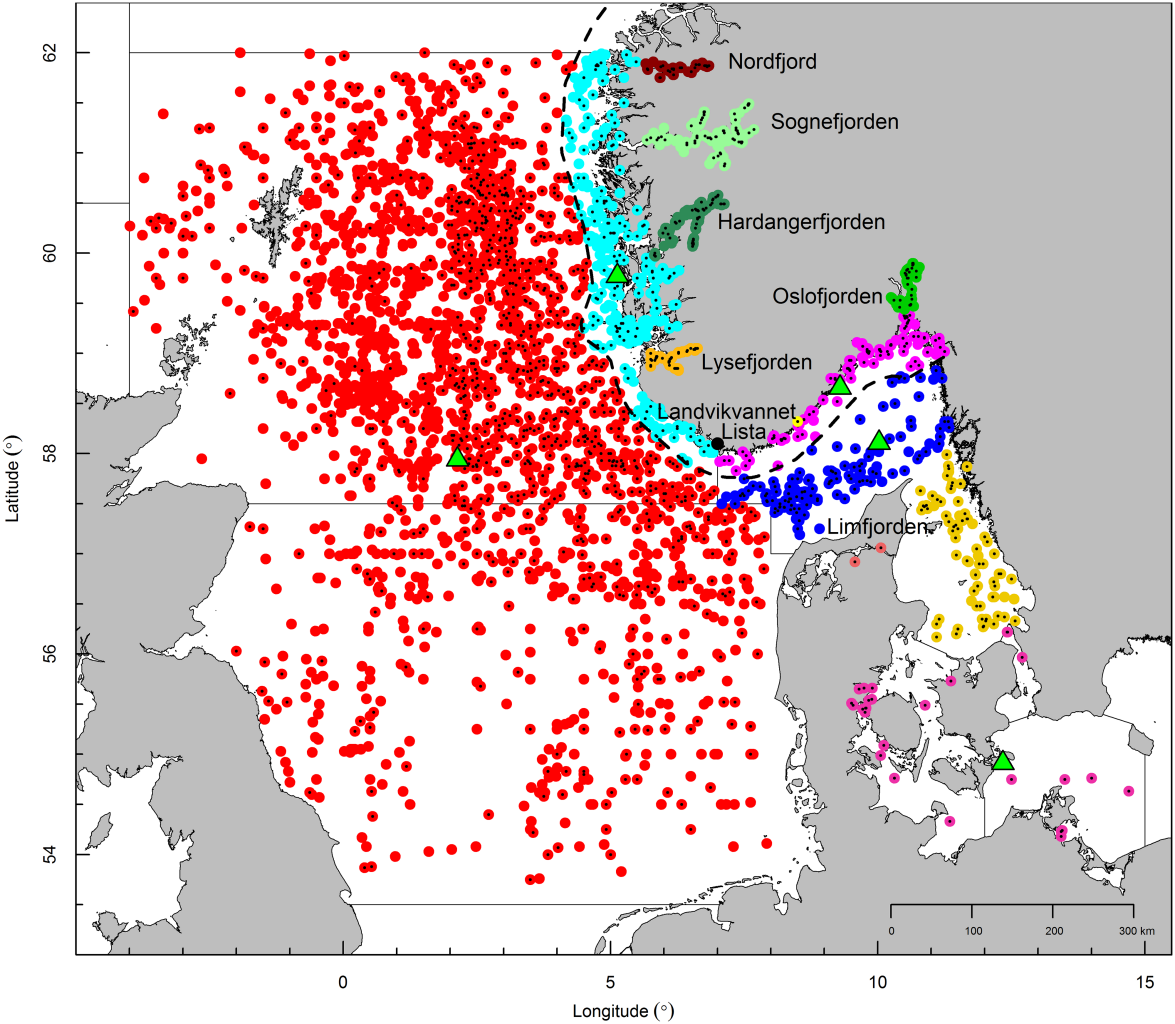
Map of the study area. Locations of sampling stations where biological data were collected from 1970–2015. There are 13 areas that include northern sections of the North Sea (red), west coast of Norway (cyan), east coast of Norway (purple), Skagerrak (blue), Kattegat (gold), western Baltic (pink) and adjacent fjords or lakes (also color-coded). Black dots indicate stations where the number of vertebrae were counted. Thick black stippled line = 12 NM zone. Mean position of hydrographic samples (green triangles) are shown for the North Sea, west coast, east coast, Skagerrak and western Baltic.

Herring (*Clupea harengus*) in the Atlantic are known for their complex population structure [3]. In the northeast Atlantic, several herring populations, which can be distinguished by spawning grounds and spawning times, otolith characteristics and number of vertebrae [e.g. 4, 5], have been identified [6, 7]. However, for management purposes, it is necessary to assign all herring catches to an appropriate stock. Herring caught in the North Sea or Skagerrak (Fig 1) are managed as three different stocks[8]. The majority of individuals are assigned to either the North Sea autumn spawners (NSAS) or the western Baltic spring spawners (WBSS). The third stock, the Norwegian spring spawners (NSS), occurs in smaller proportions along the Norwegian coast [9] and in the Skagerrak [10, 11]. The eastern North Sea and Skagerrak is known to be an important area where mixing of a number of herring population occurs [12], with important implications for fisheries management [13].

Whilst individuals are assign to one of the three main stocks (NSAS, WBSS and NSS), smaller populations which occur within the area are not assessed separately by ICES. These include smaller spring spawning populations in the North Sea [14], summer/autumn spawning populations [15] and several local fjord populations [16] along the Norwegian coast. Most local populations along the Norwegian coast encounter relatively uniform and stable environmental conditions within the fjords [17]. Other local herring populations (based on their geographical location and biological characteristics) have been identified in semi-enclosed coastal systems or even lakes. However, many of these local populations migrate at least once each year, e.g. Limfjorden [18] or Landvikvannet [10] and could occur in catches from fisheries targeting NSAS and WBSS herring. Despite some evidence of connectivity between local and offshore populations [5, 11], their interaction and connectivity have not been fully explored as yet. The maintenance of diversity by avoiding overexploitation of these local populations is an important objective in management [19, 20].

A number of individual characteristics can be used to assign or identify the population origins [4, 21, 22]. Here, we utilize the number of vertebrae as a means of distinguishing populations. Environmental conditions such as temperature and salinity can influence the number vertebrae, but it is fixed already during the embryonic development [23, 24]. However, differences in vertebral counts between populations also has a genetic basis [25]. Consequently, the range in numbers of vertebrae is population specific, which allowed Swain et al. [26] to delineated stocks of Atlantic cod (*Gadus morhua*) using vertebral counts. Herring populations have also been distinguished based on vertebral counts [11, 27], and stock composition in catches and surveys have been determined in this way for management purposes [8, 28, 29].

The main objective of this study is to investigate the complex population structure of Atlantic herring, by comparing historical data of the temporal and spatial variation in phenotypic characters of herring from 13 different geographical areas: four offshore areas (North Sea, Skagerrak, Kattegat and western Baltic), two coastal areas (west and east coast of Norway) and seven local fjords and lakes along the Norwegian and Danish coast (Fig 1). Distinct populations were identified based on phenotypic characteristics, and their maturity and potential mixing and interaction in time and space were investigated. Studies such as these are important for determining the extent of populations co-occurring in space and time, and thus being subjected to a fishery not recognizing the extent of mixed populations in catches.

## Materials and methods

### Study area

Samples from the northern North Sea, Skagerrak, Kattegat, western Baltic and southern Norwegian coast (including several fjords and lakes) were examined. To analyze spatial aspects of populations, the area was partitioned and fish were classified according to their location of capture (see Fig 1). The North Sea, Skagerrak, Kattegat and western Baltic were classified as offshore areas. The west and east coast of Norway were defined as coastal areas. These five major areas, except Kattegat due to data limitation, were included in the analyses to investigate the dynamics of maturation and spawning time and vertebral counts. A third geographical classification was the ‘local population area’ that included fjords and some small lakes, such as Nordfjord, Sognefjorden, Hardangerfjorden and Lysefjorden (along the western coast of Norway). Local population areas also included Landvikvannet, Oslofjorden (along the eastern coast of Norway) and Limfjorden (Denmark). The local population areas are adjacent to the offshore areas and potentially inhabited by herring populations. S1 Supporting Information gives a detailed description of all the areas investigated.

### Data sources

#### Biological data

Biological data from 428,773 herring, collected from the years 1970–2015, were extracted from databases at IMR (Norway) and DTU-Aqua (Denmark) and used for the analyses. All data from the IMR database were included, whereas only data with vertebral counts were extracted from the DTU-Aqua database. In general, samples from the same time and location which had less than 10 herring were excluded. The data originated both from regular scientific surveys and commercial catch sampling. The time and area of fish capture as well as the fishing gears used varied (S1–S3 Tables). Due to the survey design in the local fjords, there was a potential bias towards smaller fish in these areas. There was repeated annual sampling in fjords and it is generally assumed that the presence of older and larger fish demonstrates the presence of local spawning populations, whereas their absence would indicate that the area was a nursery area with no local herring population.

The usual standard sample size comprised 100 herring, but some samples were smaller (limited by small total catches). Biological parameters included total length (nearest 0.5 cm below), sex, stage of maturity, age (as determined by counts of winter rings (wr) from otoliths), and, for most samples, the number of vertebrae. Maturity stages were determined by visual inspections of gonads according to the following scale: immature = 1–2, maturing = 3–4, ripe = 5, spawning/running = 6, spent = 7 and recovering = 8 [30].

#### Physical data

Annual mean temperatures and salinities of sampling areas were estimated for each spawning seasons in each area. The spawning seasons were defined as August-October for the North Sea [6], February-March for the west coast [31], March-May for the east coast, Skagerrak, and the western Baltic [7, 32]. These periods correspond to the time when vertebral counts are fixed [23, 24]. Temperature and salinity data for the spawning seasons within each area were extracted from the ICES Dataset on Ocean Hydrography [33]. Values used for temperature and salinity were the means between depths of 20–150 m.

### Ethical statement

Most samples were collected during standard scientific surveys or from commercial catches in national and international waters under international rights. Otherwise, the Institute of Marine Research (IMR), which is responsible for monitoring herring and giving advice to fisheries managers in Norway, has permission to sample herring at any location along the Norwegian coast by the Directorate of Fisheries, Bergen, Norway. The same accounts for the DTU-Aqua in Denmark. A special permission to sample herring with gillnets inside Landvikvannet was granted by the County Governor of Aust-Agder, Arendal, Norway. Our study did not involve any endangered or protected species.

### Data analysis

All statistical analyses and figures were made using statistical software packages in R [34]. For all tests, we used 95% as the level of significance.

Length-at-age data, used as a proxy for growth of herring, were fitted to the von Bertalanffy growth model (VBGM) [35]:

$$L_{t}=L_{\infty }(1-e^{-K(t-t_{0})})$$

where *L*_*t*_ is the average length at age *t*, *L*_*∞*_ is the asymptotic maximum length, *K* is the von Bertalanffy growth rate coefficient (i.e., the rate at which length approaches the maximum length asymptote) and *t*_*0*_ is the intercept on the time axis.

Length-at-first-maturity was quantified by a generalized linear model [36] using a logistic regression to estimate the probability of herring to be mature at a certain length. Herring were grouped as immature or mature. The following model was used to calculate the length when 50% and 95% were mature:

$$logit(M)=\beta_{0}+\beta_{1}\times L$$

where *β*_*1*_ describes the estimated effect of length *L* in the probability *(M)* to be mature.

**Fig 2:**
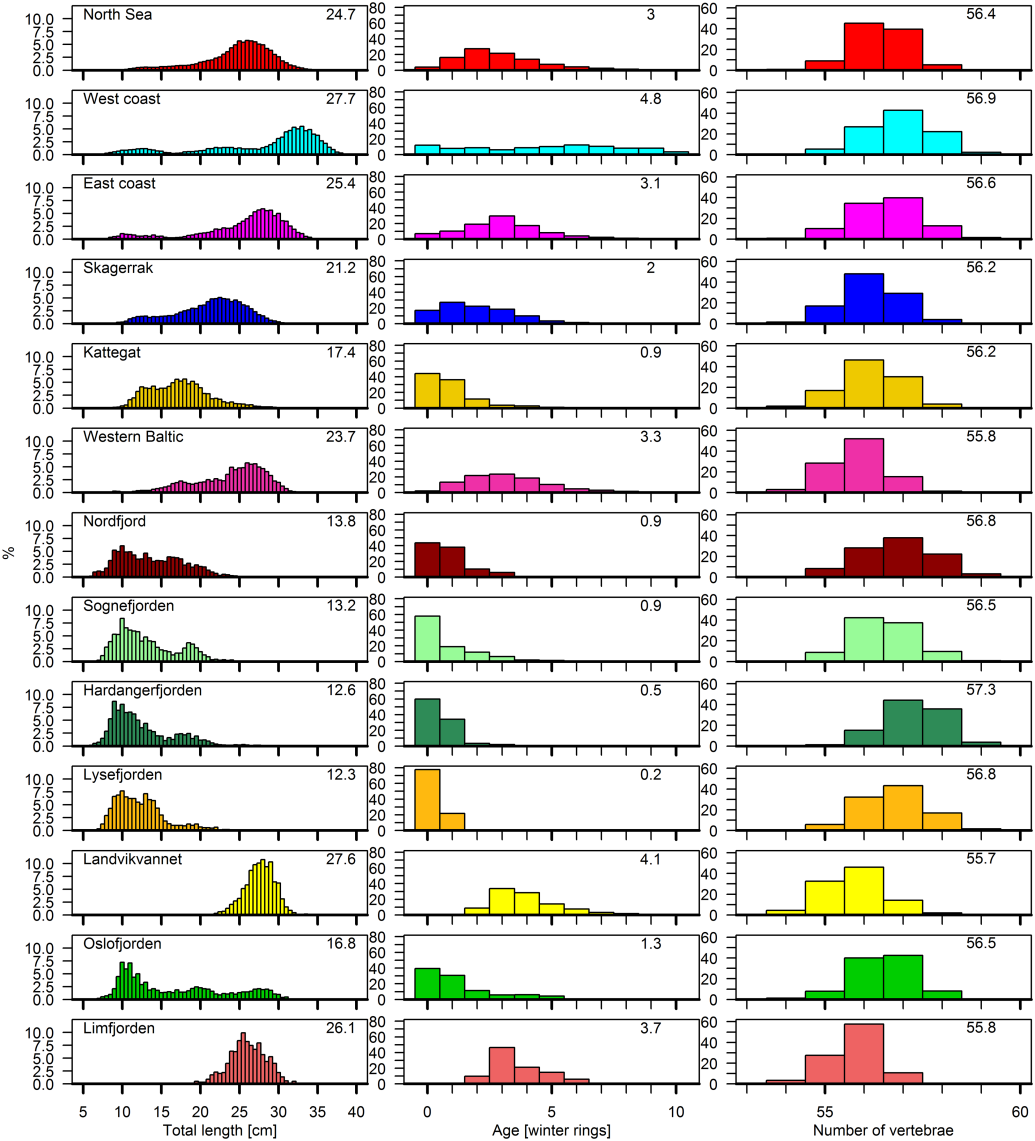
Distribution of biological characteristics. Histograms showing the total length, age and number of vertebrae (VS) by area (data from 1970–2015 pooled) including the mean value for all characters. The range of total length, age and VS was scaled to the amount of herring so very small values are not visualized.

For spatio-temporal comparison, the mean number of vertebrae (VS) was calculated for each ICES Statistical rectangle (1° Longitude, 0.5° Latitude) per quarter of the year. For purposes of illustration, these results were divided into five categories. The mean number of vertebrae of western Baltic spring spawners (<55.9) [28], North Sea autumn spawners (~56.5) [28] and Norwegian spring spawners (>57.2) [37] were used as reference points of the five categories. The variability of VS within each area was estimated as the difference of samples from the overall mean of the area (see Fig 2). In this study, a difference larger than ±0.25 was defined as highly variable (S1 Fig).

For the following analyses, only mature herring with three or more winter rings were used. In addition, only data from the five major areas (North Sea, west coast, east coast, Skagerrak and western Baltic) were included to investigate the dynamics in these areas. The data were fitted to generalized additive models (GAMs), since they allow flexible non-parametric effects of covariates [38]. Model selection was based on the generalized cross validation (GCV) score. Residual plots were used for checking model fits, and isotropic smoothing functions *s()*, uniform in all orientations, were used to define smooth terms (thin-plate regression spline) [39]. For further details on data exploration and model selection see SI Materials and methods.

The final GAM for dynamics among the areas in terms of vertebral count differences was:

$${VS}_{i}=\alpha +\beta (Area)+s({Yclass}_{i})+s({Quat}_{i})+s({Mat}_{i})+\varepsilon_{i}$$

where *VS* is the number of vertebrae, *Area* represents the five major areas, *Yclass* is the calculated year of hatching (= sampling year minus the age (wr)). For autumn spawners (assumed for the North Sea) the actual age is wr + 1 since no winter ring is formed during their first winter. Aging methods were the same in all areas and did not affect the analyses. *Quat* and *Mat* represent the quarter of the year when herring were sampled and the stage of maturity, respectively, and *ε* is the error term. Since the quarter of year and maturity stage were not correlated, differences in VS for pre-, post- or spawning herring would show the occurrence of different populations/stocks due to migration into and out of areas. Length, age, gear type, and the mean temperature and salinity during hatching of each year class were included in the initial model, but removed due to non-significance.

Spawning dynamics were analyzed by calculating the proportion of pre-spawning, spawning, and post-spawning herring per sample. For a comparison of intra-annual variations, the data of pre-spawning, spawning and post-spawning herring were fitted individually to a GAM:

$${Prop}_{i}=\alpha +\beta (Area)+s({Month}_{i})+\varepsilon_{i}$$

where *Prop* is the proportion of pre-, post- or spawning herring in percent and *Month* represents the sampling month.

## Results

### Area specific characteristics

The length and age of herring sampled ranged from 4.5–45.0 cm and 0–17 winter rings (wr). There were no significant sex differences in the biological characters analyzed in the data sets (ANCOVA; p>0.05). Therefore, all further analyses were carried out with sexes combined. Vertebral counts, the main population specific trait selected for this study, did not differ between scientific and commercial catches or fishing gears between and within each area (ANOVA; p>0.001).

A comparison among the five major areas, Kattegat and seven local fjords gave significant differences in length, age (wr) and vertebral counts (VS) (ANOVA; p_length_<0.001, p_age_<0.001, p_VS_<0.001, Fig 2). The main tendency, based on Tukey-HSD tests, was an increase in length and age going from the fjords towards offshore (North Sea, Skagerrak and Kattegat) and back to the coast (western and eastern coast of Norway), with west coast herring being the largest and oldest of all groups (Fig 2). Landvikvannet and Limfjorden had larger and older fish compared to other fjords, because no juveniles were sampled. The highest VS were found at the west coast and inside the fjords along the west coast. One exception was the herring in Sognefjorden that had intermediate VS, similar to herring collected in the North Sea, east coast and Oslofjorden. The lowest VS were observed for herring in Landvikvannet, comparable to herring from the western Baltic and Limfjorden.

For most of the areas, high variability in VS occurred (S1 Fig). The largest variance was ±1.2 from the overall mean. Within the North Sea, western Baltic, Kattegat, Landvikvannet, Oslofjorden and Limfjorden nearly all samples had a variance lower than our threshold of ‘high variability’ (±0.25).

### Body growth

The growth of herring, estimated from historical length-at-age data, differed among all areas (ANOVA; p<0.001, Panel A in S2 Fig). All coastal herring had higher growth rate than other offshore or fjord herring. Lowest growth rate occurred in Nordfjord and Sognefjorden. Fitting the historical observed length-at-age data to the von Bertalanffy growth model highlighted differences between all areas (ANOVA; p<0.001, Table 1, Panel B in S2 Fig). Again, coastal herring had the largest maximum asymptotic length (L_∞_) of all groups. Similar maximum length and growth rate (K) were observed in the offshore North Sea and western Baltic as well as in Landvikvannet, Oslo- and Limfjorden. Herring in the Skagerrak and Kattegat had the lowest maximum length among the offshore areas. However, the overall lowest maximum length was for herring in the Nordfjord and Sognefjorden.

**Table 1:** Estimated parameters ± standard error for the von Bertalanffy growth model and the generalized linear model quantifying the length-at-first-maturity for each area. Length where 50% (L50) and 95% (L95) of herring were mature and numbers of observation (N) with valid data are given for each area. Growth data from Lysefjorden was not sufficient to estimate growth model parameters. Due to missing of mature herring (Lysefjorden) and immature herring (Landvikvannet and Limfjorden), no maturity probabilities could be calculated.

	North Sea	West coast	East coast	Skagerrak	Kattegat	Western Baltic	Nordfjord	Sogne- fjorden	Hardanger- fjorden	Lyse- fjorden	Landvik- vannet	Oslo- fjorden	Limfjorden
Growth													
L_∞_	30.16±0.01	35.20±0.03	31.18±0.04	27.33±0.05	29.25±0.36	29.95±0.09	23.55±0.34	22.16±0.20	30.88±0.81	22.07±1.45	30.12±0.02	29.41±0.20	30.14±0.42
K	0.48±0.00	0.36±0.00	0.54±0.00	0.52±0.00	0.32±0.01	0.43±0.01	0.59±0.03	0.53±0.02	0.41±0.02	1.05±0.27	0.48±0.04	0.52±0.01	0.49±0.05
t_0_	-1.15±0.00	-1.17±0.01	-0.97±0.01	-1.39±0.01	-2.08±0.05	-1.01±0.02	-0.95±0.03	-1.36±0.04	-1.08±0.03	-0.71±0.11	-1.48±0.28	-0.93±0.02	-0.66±0.26
N	208 348	34 142	27 646	34 813	7 018	10 382	2 069	4 635	2 729	2 683	2 386	2 404	1 177
Maturity													
Intercept (β_0_)	-16.5±0.08	-18.1±0.28	-16.8±0.23	-14.3±0.18	-23.8±0.90	-13.1±0.27	-10.5±0.81	-14.7±0.82	-43.2±6.38			-24.9±1.51	
Length (β_1_)	0.7±0.00	0.7±0.01	0.7±0.01	0.6±0.01	1.1±0.04	0.6±0.01	0.5±0.04	0.8±0.04	1.9±0.28			1.1±0.06	
L50	23.65	24.73	24.51	25.23	22.41	21.50	19.70	17.80	23.11			23.23	
L95	27.88	28.76	28.80	30.42	25.18	26.31	25.21	21.36	24.68			25.98	
N	195 905	30 850	26 397	28 808	3 780	10 509	818	1 505	522			1 127	

### Maturity ogives

The maturity ogives of herring differed among all areas when comparing the length at 50% (L_50_) or 95% (L_95_) mature (ANOVA; p<0.001, Table 1, Panel C in S2 Fig). Herring in both coastal areas (west and east), as well as in the North Sea and Skagerrak, matured at larger body sizes compared to other areas. While 50% of herring in Oslo- and Hardangerfjorden were mature at approximately the same intermediate length, the L_95_ in Hardangerfjorden was the third smallest. Herring in Nordfjord and Sognefjorden were mature at the smallest lengths. Both western Baltic and Kattegat herring had an intermediate length-at-maturity, as compared to coastal and offshore herring (high) and fjord herring (low).

### Maturation and spawning time

**Fig 3:**
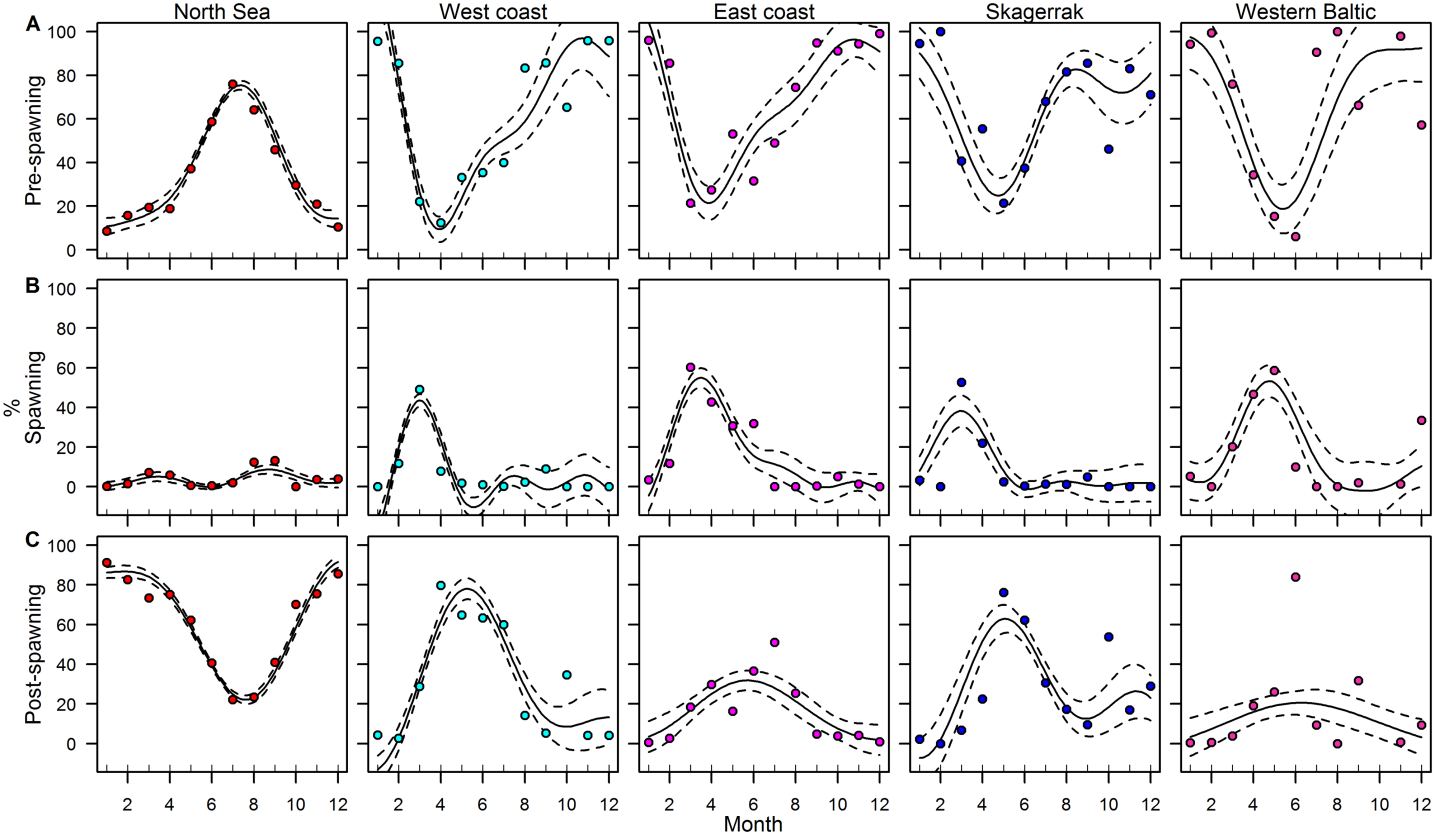
Annual variation of maturity. Fitted results for the GAMs on the proportion of pre-spawning (A), spawning (B) and post-spawning (C) herring per month. Solid lines indicate the fitted values, dashed lines the 95% confidence intervals and points the observed values for the five major areas.

Based on the distribution of spawning herring by area and month, the herring from the five major areas included in these analyses could be separated into three main groups: autumn spawners (September-October), early spring spawners (February-April) and late spring spawners (March-June) (Fig 3). The proportion of pre-, post- and spawning herring differed within each area (Table 2). Autumn spawning herring were observed in the North Sea and along the west coast. Early spring spawners occurred solely in the west coast area, whereas late spring spawners dominated the east coast area, Skagerrak and western Baltic. In the North Sea, a small proportion of spawning herring also occurred in early spring.

**Table 2:** Estimates of GAMs for the effects on vertebral counts and the monthly spawning dynamics in terms of the proportion of pre-, post- and spawning herring. Modeled means together with each covariate’s degrees of freedom (Ref.df, explaining the oscillations of the modeled trend, where 1 would be linear) and p-value for each area, and the deviance explained (R^2^) by the model, are shown. For temperature and salinity, estimates were given before the variables were dropped from the model.

Model	North Sea			West coast			East coast			Skagerrak			Western Baltic			R^2^ (%)
	Mean	Ref.df	p-value	Mean	Ref.df	p-value	Mean	Ref.df	p-value	Mean	Ref.df	p-value	Mean	Ref.df	p-value	
Vertebral counts																
Year class	56.5	8.6	<0.001	56.9	8.9	<0.001	56.6	9.0	<0.001	56.1	7.2	<0.001	55.9	5.5	<0.01	17.7
Quarter of the year		3.0	<0.001		3.0	<0.001		3.0	<0.001		2.9	<0.001		2.2	<0.001	
Stage of maturity		5.0	<0.001		4.6	<0.001		5.0	<0.001		4.8	<0.001		1.0	<0.001	
Temperature		2.6	0.18		5.6	0.06		5.3	0.54		4.5	0.08		1,0	0.05	
Salinity		1.5	0.43		6.4	0.07		1.0	0.75		1.1	0.69		1.0	0.05	
Spawning dynamics																
Pre-spawning	38.5	7.0	<0.001	61.6	7.0	<0.001	66.0	7.0	<0.001	65.8	6.6	<0.001	62.1	6.4	<0.001	38.4
Spawning	2.4	8.5	<0.001	4.5	9.0	<0.001	15.8	8.9	<0.001	7.7	8.3	<0.001	16.7	8.3	<0.001	39.8
Post-spawning	59.5	5.0	<0.001	34.9	5.0	<0.001	17.0	4.7	<0.001	28.6	5.0	<0.001	12.1	3.2	0.04	51.3

The intra-annual variation of pre-spawning herring followed the opposite trend of spawning herring, with peak proportion shortly before spawning occurs. In the North Sea, very few pre-spawning herring occurred during spring, and the highest proportion was found in June-August. The changes for post-spawning herring were also related to the observed spawning time for each area. Along the western and eastern coast of Norway, high proportions of post-spawning herring were observed from April-July. In the western Baltic, barely any post-spawning herring were observed, except after the main spawning in June.

### Population mixing

**Fig 4:**
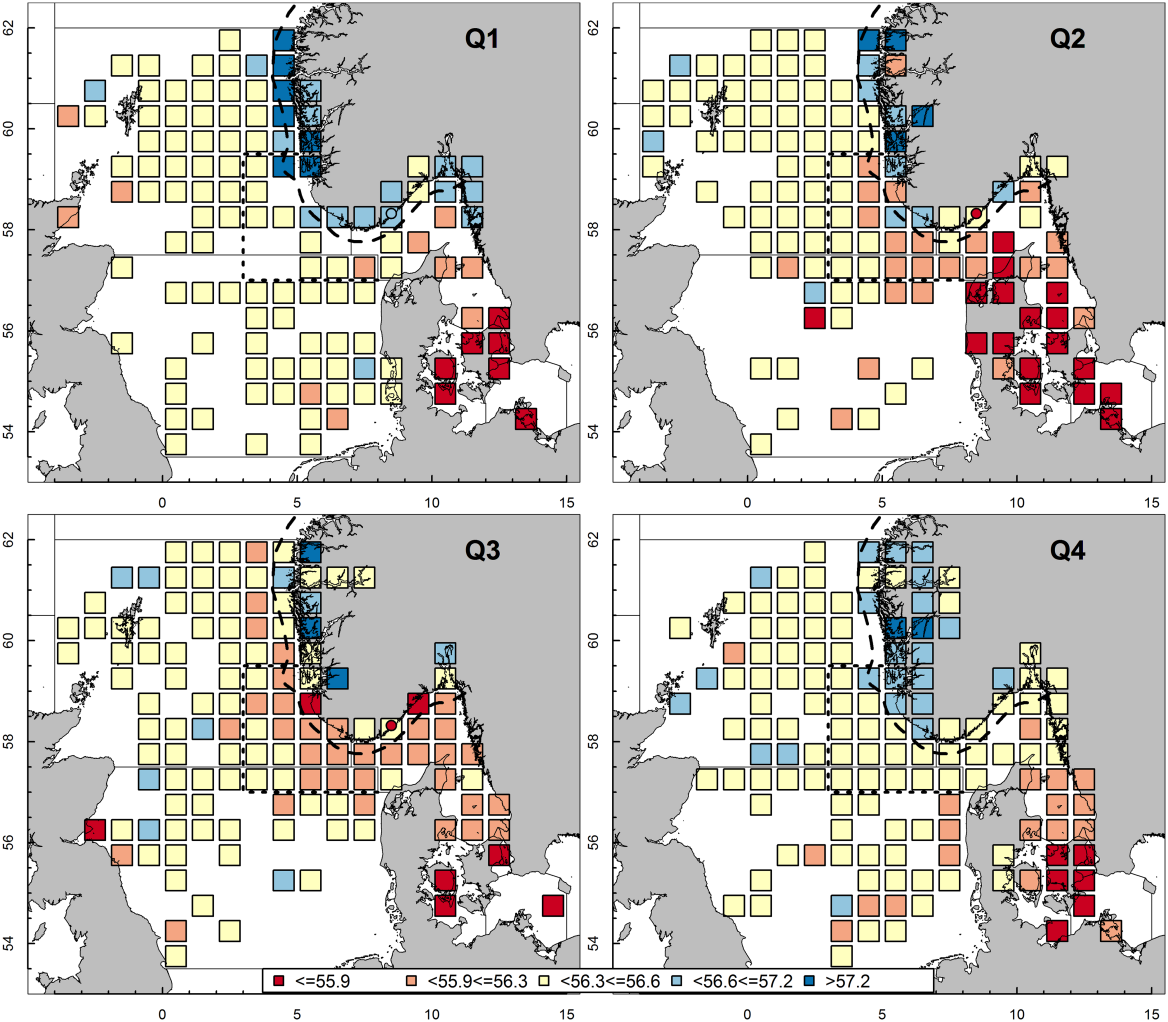
Spatial and temporal dynamics of mean vertebral counts. Mean vertebral counts (colored) for each geographic square (1° Longitude, 0.5° Latitude) and Landvikvannet (circle) per quarter (Q) of the year. ICES ‘transfer area’ is indicated by stippled line.

VS, the main population specific trait, were highly variable over the different quarters of a year in the coastal areas, eastern North Sea and Skagerrak (Fig 4). The VS of herring in the central North Sea was relatively stable throughout the year. Most variation occurred along the coast and the border between the North Sea and the Skagerrak. On the west coast, there was a clear decrease from VS 57.2 or higher in the first quarter to 55.9–56.3 or lower in the third quarter. A similar decrease occurred on the east coast and the local area of Landvikvannet, but VS were never higher than 57.2. All herring migrated out from Landvikvannet by the end of the third quarter, as evidenced by fishing efforts resulting in zero catches. In Skagerrak and Kattegat, herring with low VS occurred in the second quarter and dispersed into the eastern North Sea and up along the Norwegian west coast in the third quarter of the year (Fig 4). In the fourth quarter, the migratory populations with very high VS along the west coast and low VS in the Skagerrak and eastern North Sea disappeared and the VS became similar to the overall historic mean of these regions (see Fig 2). In the western Baltic, the low VS (55.9) was also stable throughout the year. Local fjords were not sampled continuously and consequently, VS data are sparse from those areas.

**Fig 5:**
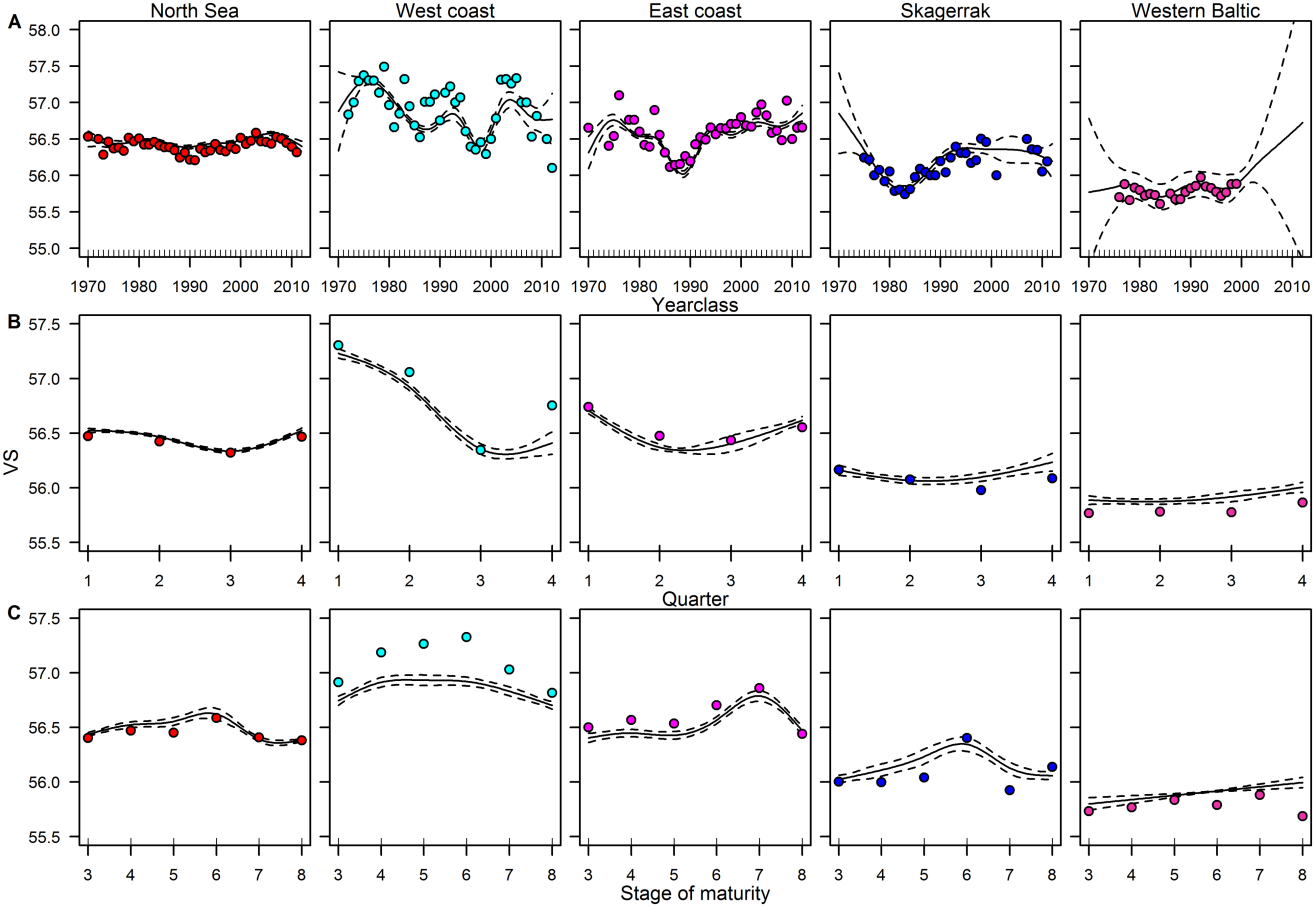
Variation and dynamic of mean vertebral counts. Fitted results for the GAM on mean vertebral counts (VS) in relation to year classes (A), quarters of the year (B) and stages of maturity (C). Solid lines indicate the fitted values, dashed lines the 95% confidence intervals and points the observed values for five major areas.

The GAM indicated significant variation of VS within each of the five major areas with respect to year class, quarter of the year and maturity stage (Fig 5; Table 2). Neither temperature nor salinity had a significant effect on the variation over time in VS. According to the GAM, VS in the North Sea and western Baltic varied significantly among different year classes, but still the variation was relatively stable around their general means of 56.4 and 55.8, respectively (Fig 5A), compared to the coastal areas and Skagerrak. Highest variation in VS occurred along the west coast; two periods with maximum VS were observed for the year classes around 1975 and 2005, while there was one period with minimal VS around 1997. At the east coast, maximum VS was found for the same periods as the west coast, but VS was only slightly higher than the overall mean. The year classes around 1990 had comparably very low VS after which it increased continuously. In the Skagerrak, VS have been stable for the last 25 years, after a minimum observed around year classes of 1983.

In addition to the inter-annual dynamics, seasonal dynamics could be seen in all areas (Fig 5B; Table 2). The highest seasonal dynamics were observed along the west coast, where VS decreased rapidly from 57.2 in the 1^st^ quarter to 56.5 in the 3^rd^ quarter, with a slight increase in the 4^th^ quarter. In comparison, VS in the other areas were relatively stable with a maximum range of 0.4. However, the general trend was a decrease in VS with a minimum in the 2^nd^ quarter (east coast, Skagerrak) or 2^nd^– 3^rd^ quarter (North Sea) followed by an increase to the same level as observed in the 1^st^ and in the 4^th^ quarter of the year.

VS also varied for different stages of maturity in all areas (Fig 5C, Table 2). In general, spawning herring had the highest VS in all areas, except the western Baltic. In the western Baltic, VS was rather stable. In the North Sea, spawning herring only had slightly higher VS than other maturity stages. Along the east coast, spent herring (stage 7) had the highest VS.

A comparison of spawning herring solely indicated stable VS among the year classes in the Skagerrak and western Baltic. During the first quarter in the North Sea, spawning herring with VS above the general mean had been observed for the last 15 years (S3 Fig). Since 1990, an increasing VS during spawning in the first quarter was observed for the west and east coast areas.

## Discussion

Analyses of historical data on the temporal and spatial variation in phenotypic characters of Atlantic herring demonstrate significant differences among 13 geographical areas. In some areas, vertebral counts (VS), the main population specific trait used in this study, are highly variable over time. Such variation indicates temporal changes in presence or absence of herring populations in an area. The temporal variation in VS was not related to environmental factors, but such factors can influence the development of meristic characters (e.g. VS) during the incubation period and early larval life [23, 24]. Therefore, one explanation for varying VS could be the co-occurrence of different herring populations in time and space, but without interbreeding. Alternatively, if herring from one population joined and interbreed with herring from another population, we would expect to see offspring with intermediate vertebral counts [26, 40].

Theoretically, the number of vertebrae is negatively correlated with temperature and positively with salinity conditions during the early larval life before vertebrae are developed [23, 24]. However, such a correlation was not apparent in our data. Also, the monotonic increase in temperature, during the study period, did not appear to have a direct influence on the number of vertebrae. Environmental factors can influence meristic characteristics, as demonstrated in laboratory studies. However, these may be masked by other factors resulting in differing vertebral counts in wild populations.

The historical North Sea data, showing stable vertebral counts over many decades, indicate no mixing of populations (Fig 5). One exception is the ‘transfer area’, as defined by ICES [8] (in the North Sea: east of 3°E and between 57–59.5°N, Fig 4), where herring with lower vertebral counts occur during the 2^nd^ and 3^rd^ quarter of the year. These herring are in pre-spawning condition and are presumably western Baltic spring spawners (WBSS) on their feeding migration [12]. Consequently, no connectivity between WBSS and North Sea herring is assumed. Mariani et al. [41] suggest that ‘isolation-by-distance’ would account for the North Sea herring being a homogenous population, genetically different from spawning aggregations in the English Channel (Downs) and along the south coast of Norway. Further, morphological differences between spawning herring in the Downs and the North Sea have been demonstrated [42] However, Downs herring, migrating into the North Sea during summer for feeding, could not in our study be identified based on biological characteristics in the North Sea. After excluding migrating WBBS herring, assessing North Sea herring as one stock, the North Sea autumn spawners (NSAS), appears reasonable. Changes in relative importance of the individual populations within a stock, like the potential increase in the Downs, could influence the perception of stock dynamics and thus management [8].

Even though the vertebral counts of herring in the North Sea have remained fairly stable, there are indications of mature herring with slightly higher VS in the 1^st^ quarter of the year (S3 Fig). These herring have the characteristics of the Norwegian spring spawning (NSS) population, which has undergone dramatic changes in stock size and major shifts in migration routes [9, 43]. Prior to the collapse of NSS in the late 1960s, herring migrated from their wintering areas along the southern border of the east Icelandic Current towards the Norwegian coast [44]. After the stock collapse, NSS were confined to the coastal areas of western Norway. After the recovery, the feeding migration extended further offshore to the more central part of the Norwegian Sea [31] and the stock also returned to their traditional spawning grounds south of 62°N [45, 46]. In our results, the presence of spring spawners (March-April) in the North Sea(Fig 3) might be an indication of NSS migrating through the North Sea once again, although it is unclear whether these are periodic nomads of NSS or resident North Sea spring spawners [47].

Similar to the North Sea area, herring in the western Baltic area constituted a single population. In this area, the number of vertebrae were stable over many decades (Figs 4 and 5). It is therefore assumed that herring in this area were fish managed as WBSS. However, in recent years, an increasing fraction of herring from the central Baltic has migrated further westwards into the western Baltic [48, 49]. Herring from the central Baltic have an even lower VS [50]. However, our data do not suggest a decrease in VS associated with western ingress of central Baltic herring into the western Baltic.

The data from Skagerrak clearly revealed a mixture of several populations, indicated by highly dynamic intermediate VS, both within a year and inter-annually (Fig 5). These populations mainly represent mixtures of herring managed as NSAS and WBSS. NSAS dominated during the first quarter of a year, followed by an increased occurrence of WBSS in summer. Further, the observed dynamics in VS could also include a mixing of local spring spawners, which migrate into the Skagerrak for feeding, together with NSAS and WBSS during spring and summer [18, 45]. The occurrence of WBSS during summer can be traced to the North Sea, and even further north along the west coast of Norway (Fig 4). Our results support the assumption that all spring spawning herring caught after the 1^st^ quarter of the year in the ‘transfer area’ are predominantly WBSS. Further, our data indicated the occurrence of different spring spawning populations in the Skagerrak and Kattegat, based on different growth rates and maturity ogives (Table 1), compared with herring in the western Baltic. These local populations originate from various spawning grounds along the Skagerrak and Kattegat coastal areas and are genetically distinct [51].

Extensive population mixing was also apparent through the high variability in VS. This indicates variability in the presence or absence of herring populations, especially during the spawning season and along the southern coast of Norway (Fig 5). There were differences in growth, length and VS between herring of the east and west coast (Fig 2 and Table 1). However, similar trends in inter-annual changes in these parameters suggest that migratory herring with higher VS occur regularly in coastal areas during spring. The migrating herring are most likely NSS, while the second population may be more stationary, coastal spring spawning populations with lower VS [11]. The occurrence of herring with high VS (above 57.0) combined with high growth along the west coast (Fig 4), is normally an indication of migratory NSS entering the area [37]. NSS reappeared at their traditional spawning grounds south of 62°N in 1989 [45, 52, 53]. This is consistent with our results showing high VS during spawning along the west coast in the 1990s (S3 Fig). In addition, the VS along the east coast also increased after 1989 during the spawning season of NSS (S3 Fig), indicating that the migration of a proportion of NSS may have continued south and eastward into the Skagerrak area. However, whilst there have been years with low VS along the west coast, indicating a higher proportion of WBSS migrating further out of the Skagerrak, the proportion of NSS along the east coast has steadily increased. The lack of variability in the vertebral counts along the east coast indicate that a small proportion of WBSS migrate to this area. The majority of WBSS stayed in the Skagerrak during their feeding migration (Fig 4), as shown by Clausen et al. [12].

In addition to the relatively large migratory herring populations, there is the evidence of distinct local and stationary populations in Kattegat, Skagerrak and the coast of southern Norway [16, 18]. These populations differ in biological characters compared to populations in the adjacent coastal areas, with for instance the presence of older individuals in Nordfjord, Sognefjorden, Landvikvannet and Limfjorden (Fig 2 and Table 1). In contrast, Hardangerfjorden and Lysefjorden seem to lack resident local populations, since there is an absence of older individuals. Lysefjorden appears to be a nursery area (only 0-1wr individuals) for mainly NSAS and small proportions of NSS according to VS, whereas Hardangerfjorden is clearly a nursery area for NSS. The historic data from Oslofjorden indicate a mixture of several populations. However, similar vertebral counts do not necessarily mean lack of a local population. Limfjorden is known to be genetically different from other local populations in the Skagerrak and Kattegat area [18] (Table 1). Our data indicate only one local population within Sognefjorden, even though two local populations were identified in the past: the Lusterfjord herring [54] and the Østerbø herring [55]. The VS in Sognefjorden indicate almost no occurrence of NSS, despite the high abundance of young-of-the-year. The small size-at-maturity and maximum length suggest the occurrence of at least one local population (Table 1). Similar results have been observed in Nordfjord, although these are not as clear as in Sognefjorden. Libungan et al. [56] demonstrated differences in otolith shape for herring from these fjords, supporting our notion of local populations. According to VS, Nordfjord is also a nursery area for NSS. Landvikvannet is completely different from the previous described local areas with much lower VS (Fig 2). No immature individuals have been observed and the local population within Landvikvannet is not stationary. Landvik herring only occur during spawning time and leave the brackish lake afterwards. This is supported by recent studies demonstrating differences in both vertebral counts [11], behavior [10] as well as genetics [16] in Landvik herring from its neighboring populations. However, local populations are included in the management of NSAS, NSS and WBSS stocks, without knowledge of the extent of mixing. Further, assessing and managing stocks close to areas inhabiting local populations is challenging even though combining biological characteristics demonstrated clear differences. This combination of biological characters can be used to identify areas with local populations and establish management regulations to ensure the maintenance and diversity of these local populations.

Despite the clear differences in VS between the areas, the variability did not allow for a clear separation of populations when mixing occurs. When only two populations are present, a discrimination in terms of proportion should be possible based on VS. However, no individual assignment to a population or even to a stock is possible by the number of vertebrae alone. Herring can be discriminated by spawning season dependent on otolith microstructure [4]. This is used currently in the study area to distinguish between NSAS and WBSS, but local populations are neglected.

Our results of stable VS, such as noted for the North Sea or western Baltic (Fig 5), might not be indicative of a single population. However, there could be a relatively stable mixture of multiple populations that could be comprised and managed as a single stock. In the North Sea for example, multiple populations have previously been defined by distinct spawning times and sites [57]. Further, these populations can be discriminated by small-scale differences in VS [27]. However, these small-scale differences could not be detected in our data, even though the high total sample size (N = 428 773) should allow for detection of small differences. The large sample size influences the deviance explained of the generalized additive model on the number of vertebrae, which was only 17%. The large sample size and use of individual vertebral counts (S8 Fig) breaks down the theory of the deviance explained for this type of model. Using individual vertebral counts resulted in similar ranges among the five major areas and no pattern was visible. Applying the GAM to mean vertebral counts per sample (S9 Fig) would increase the deviance explained, but not influence the significance of explanatory variables. However, a low deviance explained cannot necessarily be considered to be evidence of a poor fit [see pp. 118–119, 36].

In summary, the changes in VS, growth and maturity ogives observed in the extensive time series used in this study were independent of environmental effects such as salinity and temperature. Hence, along the south coast of Norway a clear mixture of more stationary coastal populations with lower VS and migratory herring with higher VS occurs during spawning. Such an overlap is a prerequisite for potential connectivity and interbreeding of populations [13], although no direct evidence for interbreeding exists in this study. High temporal variability in VS indicates mixing of herring from two or more populations and variation in intra-annular changes in their presence or absence. This mixing of populations should be considered when managing herring in this area. However, existing methods for assignment of individual herring to a population are in progress and need to be further developed.

## Supporting information

S1 Fig Variability of mean vertebral counts (VS) demonstrated by (left panel) the distribution of the standard deviation for each sample and (right panel) the variance for each sample from the mean VS of each area showed in the figure. Vertical stippled lines indicate ±0.25 variances from the mean which are defined as expected variation within each area.
(TIF)

S2 Fig Length-at-age, estimated von Bertalanffy growth models and maturity ogives. Length-at-age (A), estimated von Bertalanffy growth models (B) and maturity ogives (C, proportion of mature herring at length) by area. Points and T-bars show the mean and the 95% confidence interval. Stippled and dotted lines indicate L_50_ and L_95_, respectively, where 50% or 95% of the herring were mature. The legends are ordered according to the maximum asymptotic length or increasing L_50_. Lysefjorden is not included in the estimation of the von Bertalanffy growth model because only data for age 0–1 winter rings were available. No complete data available for maturity ogives from Landvikvannet, Lysefjorden and Limfjorden.
(TIF)

S3 Fig Mean number of vertebrae (VS) per year class for spawning herring caught in the 1^st^ quarter of the year (A) in the North Sea, (B) west coast and (C) east coast. Only areas with significant differences were shown. Horizontal lines indicate mean VS for three herring stocks in the study area, stippled = western Baltic spring spawners, solid = North Sea autumn spawners, and dotted = Norwegian spring spawners.
(TIF)

S4 Fig Validation plots for generalized additive model analyzing the number of vertebrae. See Table 1 for estimated parameters.
(TIF)

S5 Fig Validation plots for generalized additive model analyzing the proportion of pre-spawning herring. See Table 1 for estimated parameters.
(TIF)

S6 Fig Validation plots for generalized additive model analyzing the proportion of spawning herring. See Table 1 for estimated parameters.
(TIF)

S7 Fig Validation plots for generalized additive model analyzing the proportion of post-spawning herring. See Table 1 for estimated parameters.
(TIF)

S8 Fig Raw data of individual vertebrae counts (VS) of herring for each area used in the generalized additive model (GAM) analysis. The low explained variance of only 17% for the GAM is resulting from the similar range and variance of vertebrae counts for the different areas (red = North Sea, cyan = west coast, purple = east coast, blue = Skagerrak, pink = western Baltic).
(TIF)

S9 Fig Mean vertebrae counts (VS) for each herring sample for each area. This data was not used in the generalized additive model (GAM) analysis, but would have increased the low explained variance of only 17% for the GAM, because the range and variance of vertebrae counts differs for the five areas (red = North Sea, cyan = west coast, purple = east coast, blue = Skagerrak, pink = western Baltic).
(TIF)

S1 Supporting Information Further details on material and methods. (PDF)

S1 Table Total number of analyzed herring per year per area 1970–2015. (PDF)

S2 Table Total number of analyzed herring per month per area 1970–2015. (PDF)

S3 Table Total number of analyzed herring by fishing gear per area 1970–2015. (PDF)
