# Supplementary figures and images for: Comparative biology and population mixing among local, coastal and offshore Atlantic herring (Clupea harengus) in the North Sea, Skagerrak, Kattegat and western Baltic
Source: PLoS One. 2017 Oct 30;12(10):e0187374. doi: 10.1371/journal.pone.0187374 (PMC5662228; doi:10.1371/journal.pone.0187374)

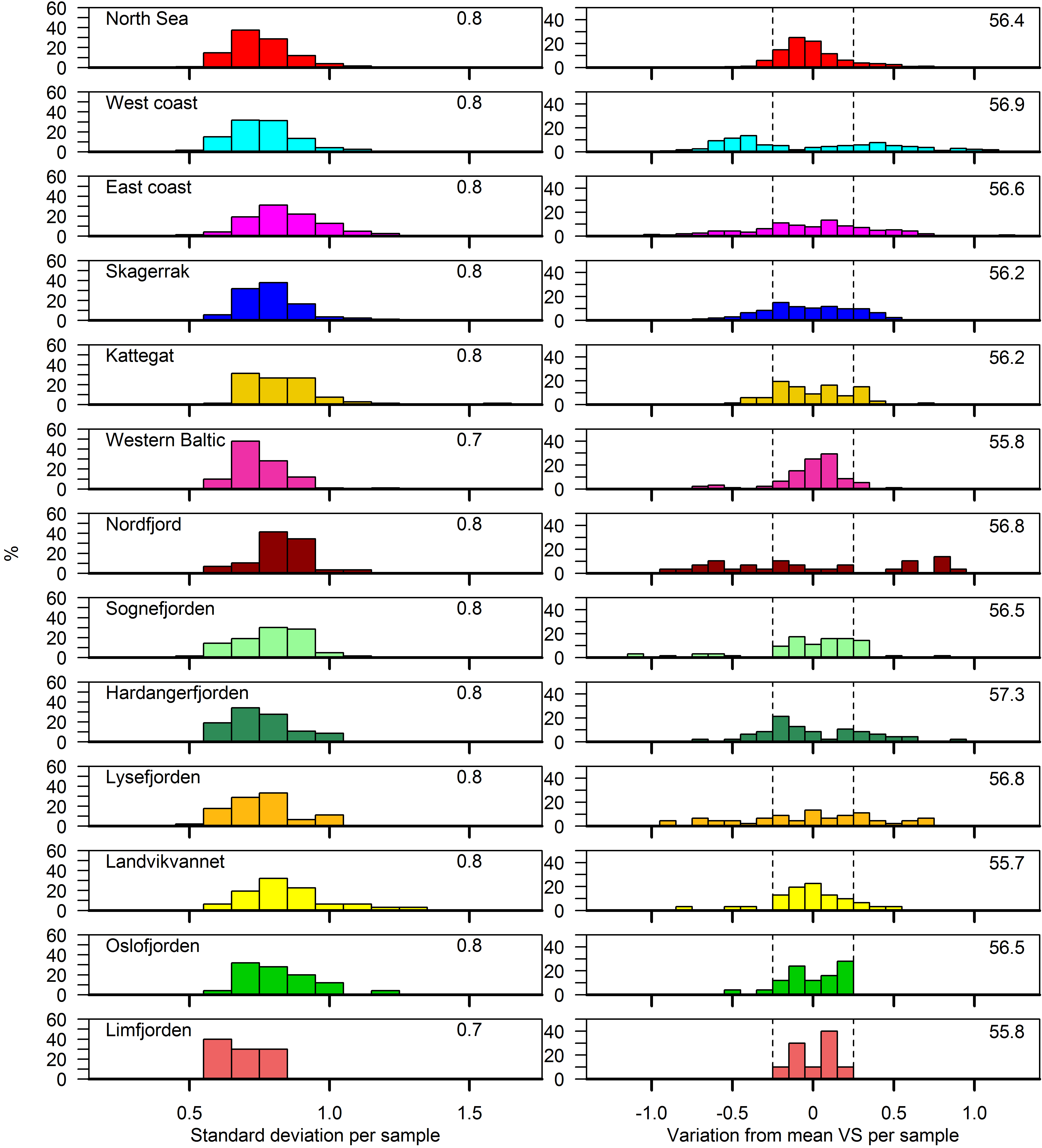

Supplement: S1 Fig — Vertical stippled lines indicate ±0.25 variances from the mean which are defined as expected variation within each area. (TIF) [file pone.0187374.s001.tif]

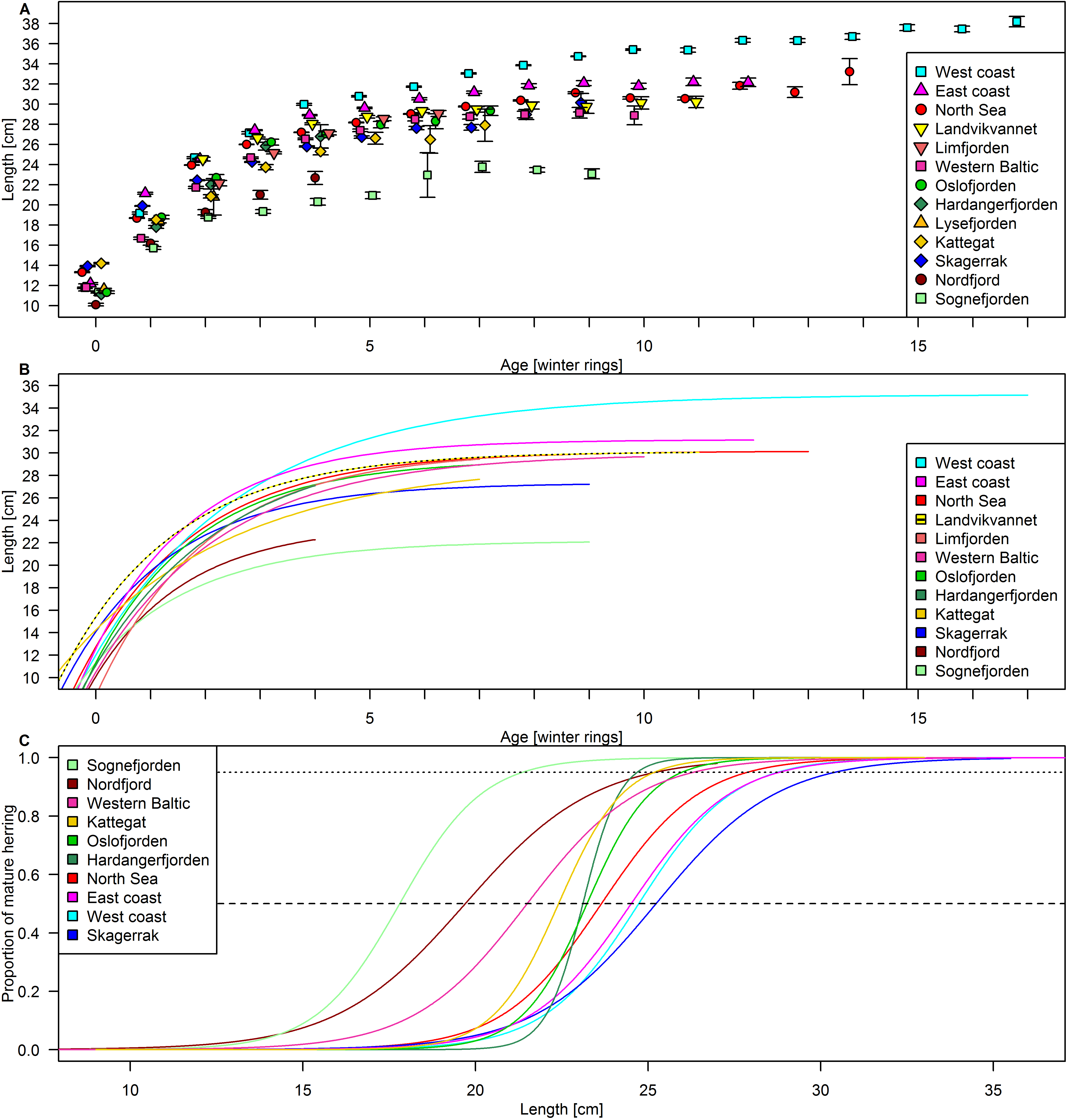

Supplement: S2 Fig — Length-at-age (A), estimated von Bertalanffy growth models (B) and maturity ogives (C, proportion of mature herring at length) by area. Points and T-bars show the mean and the 95% confidence interval. Stippled and dotted lines indicate L50 and L95, respectively, where 50% or 95% of the herring were mature. The legends are ordered according to the maximum asymptotic length or increasing L50. Lysefjorden is not included in the estimation of the von Bertalanffy growth model because only data for age 0–1 winter rings were available. No complete data available for maturity ogives from Landvikvannet, Lysefjorden and Limfjorden. (TIF) [file pone.0187374.s002.tif]

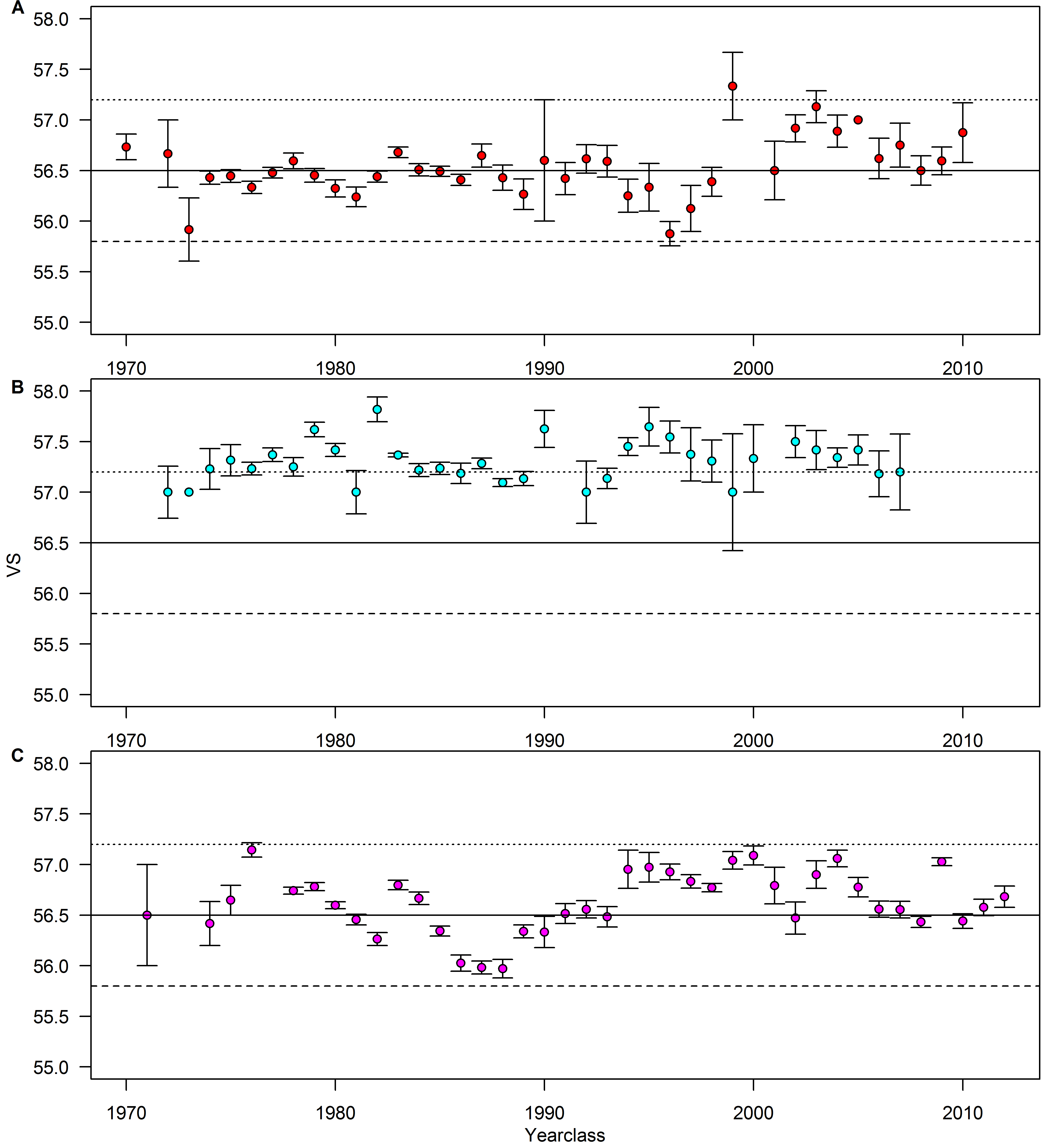

Supplement: S3 Fig — Only areas with significant differences were shown. Horizontal lines indicate mean VS for three herring stocks in the study area, stippled = western Baltic spring spawners, solid = North Sea autumn spawners, and dotted = Norwegian spring spawners. (TIF) [file pone.0187374.s003.tif]

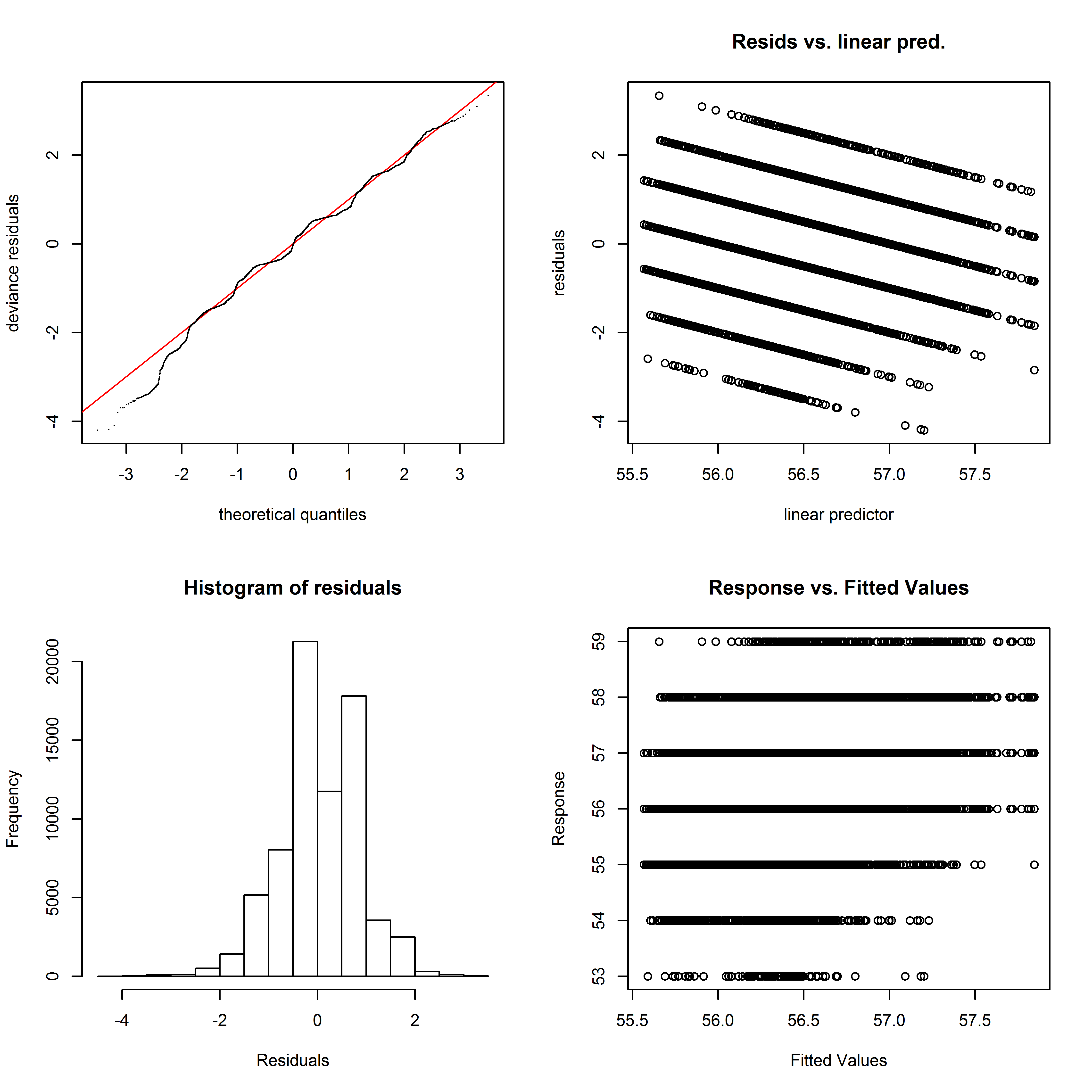

Supplement: S4 Fig — See Table 1 for estimated parameters. (TIF) [file pone.0187374.s004.tif]

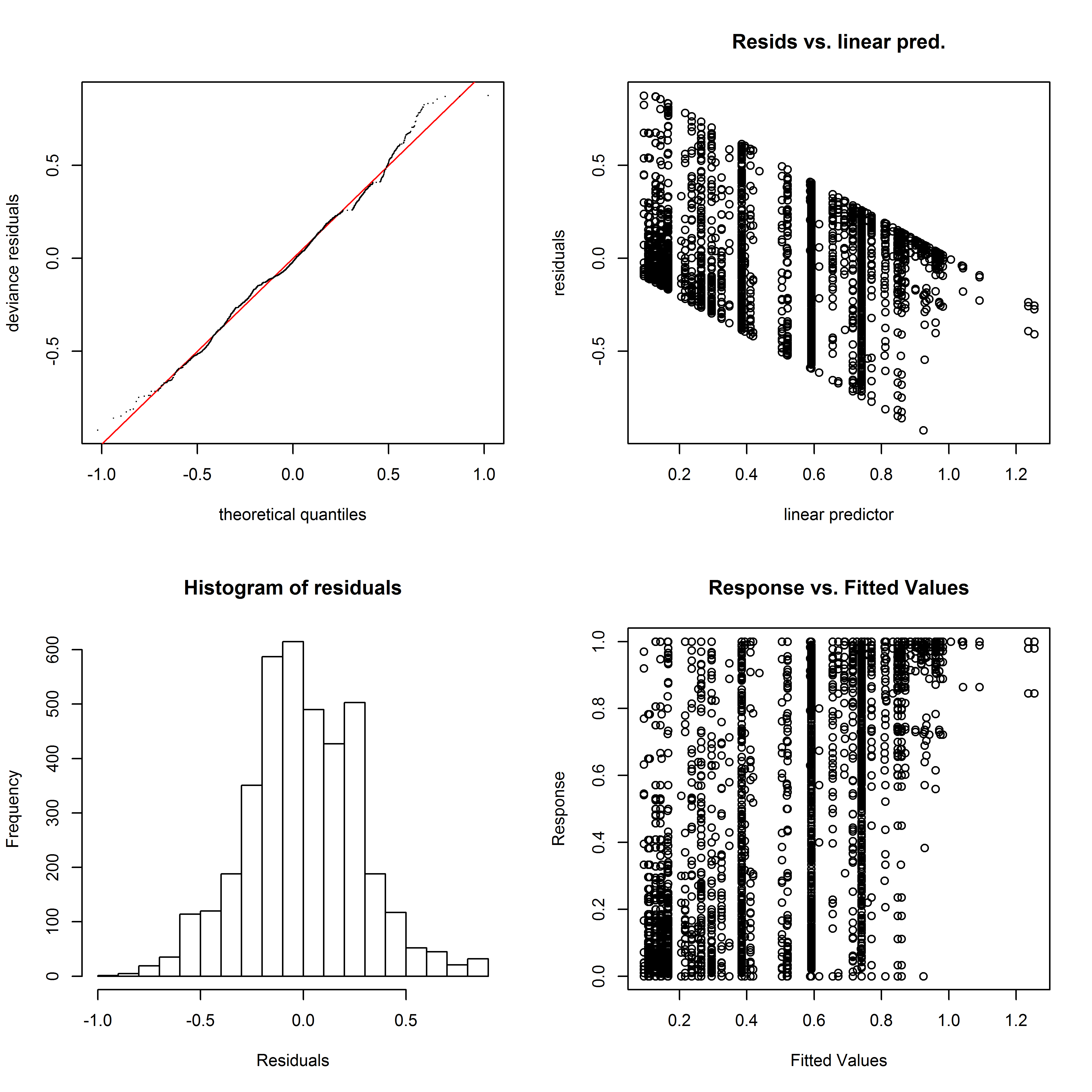

Supplement: S5 Fig — See Table 1 for estimated parameters. (TIF) [file pone.0187374.s005.tif]

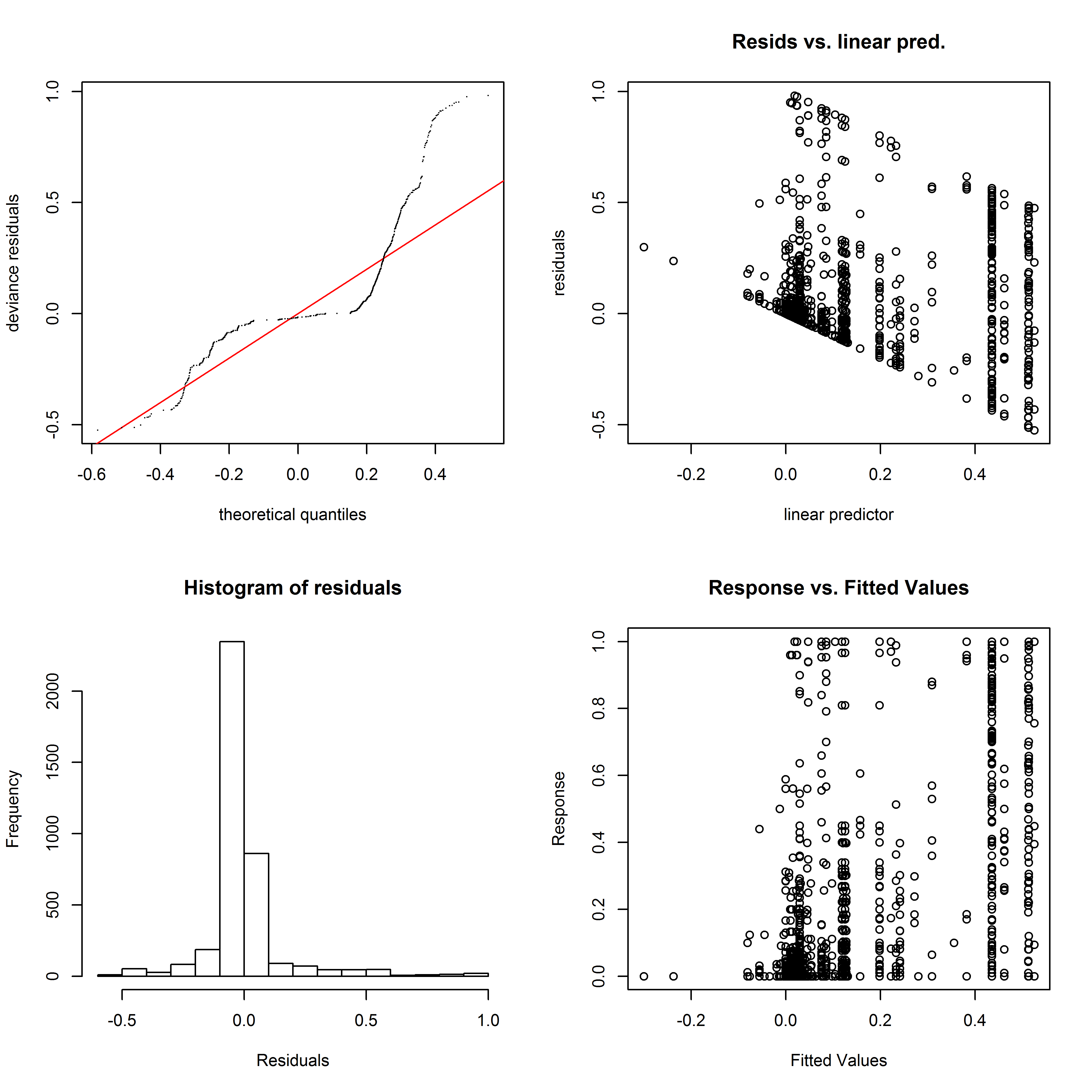

Supplement: S6 Fig — See Table 1 for estimated parameters. (TIF) [file pone.0187374.s006.tif]

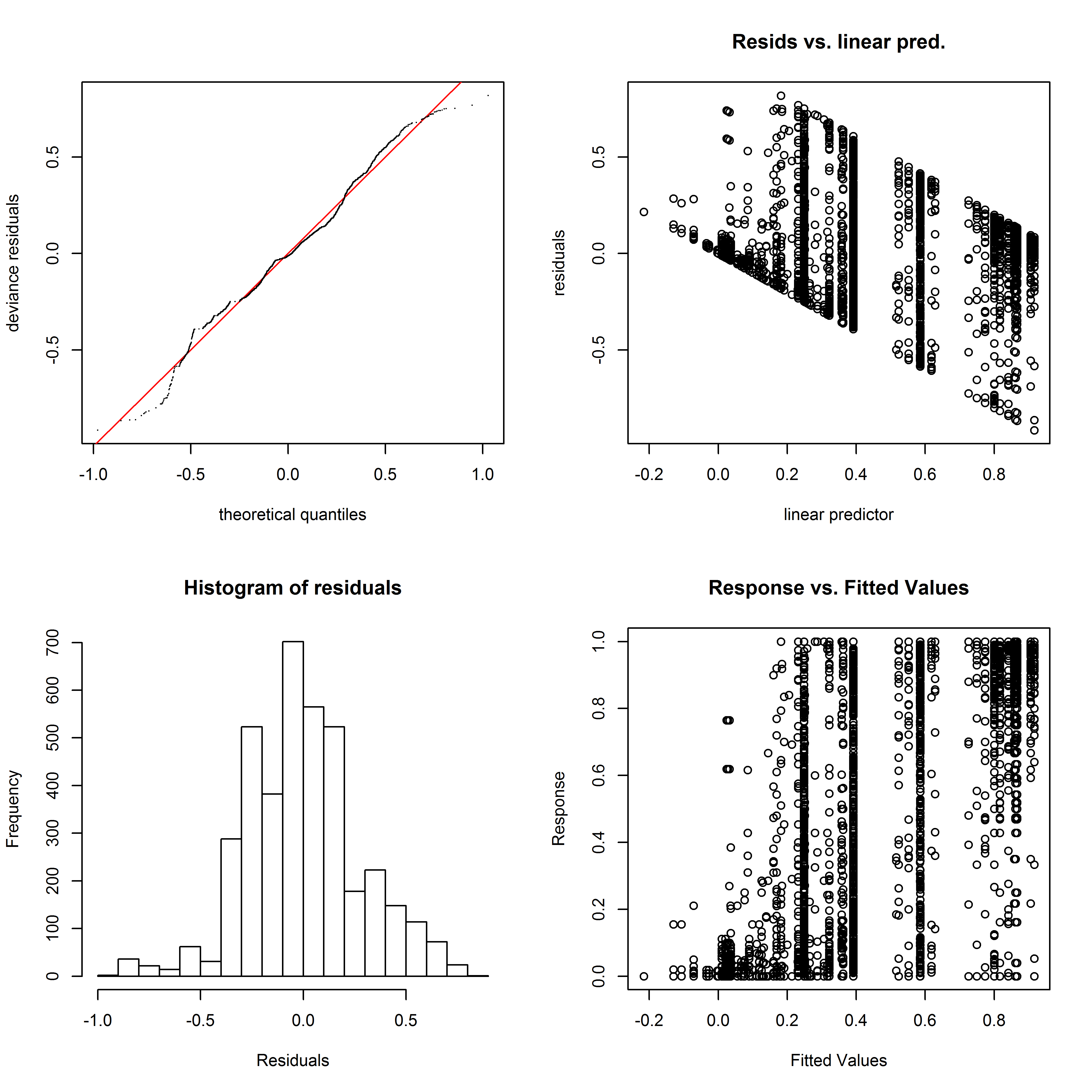

Supplement: S7 Fig — See Table 1 for estimated parameters. (TIF) [file pone.0187374.s007.tif]

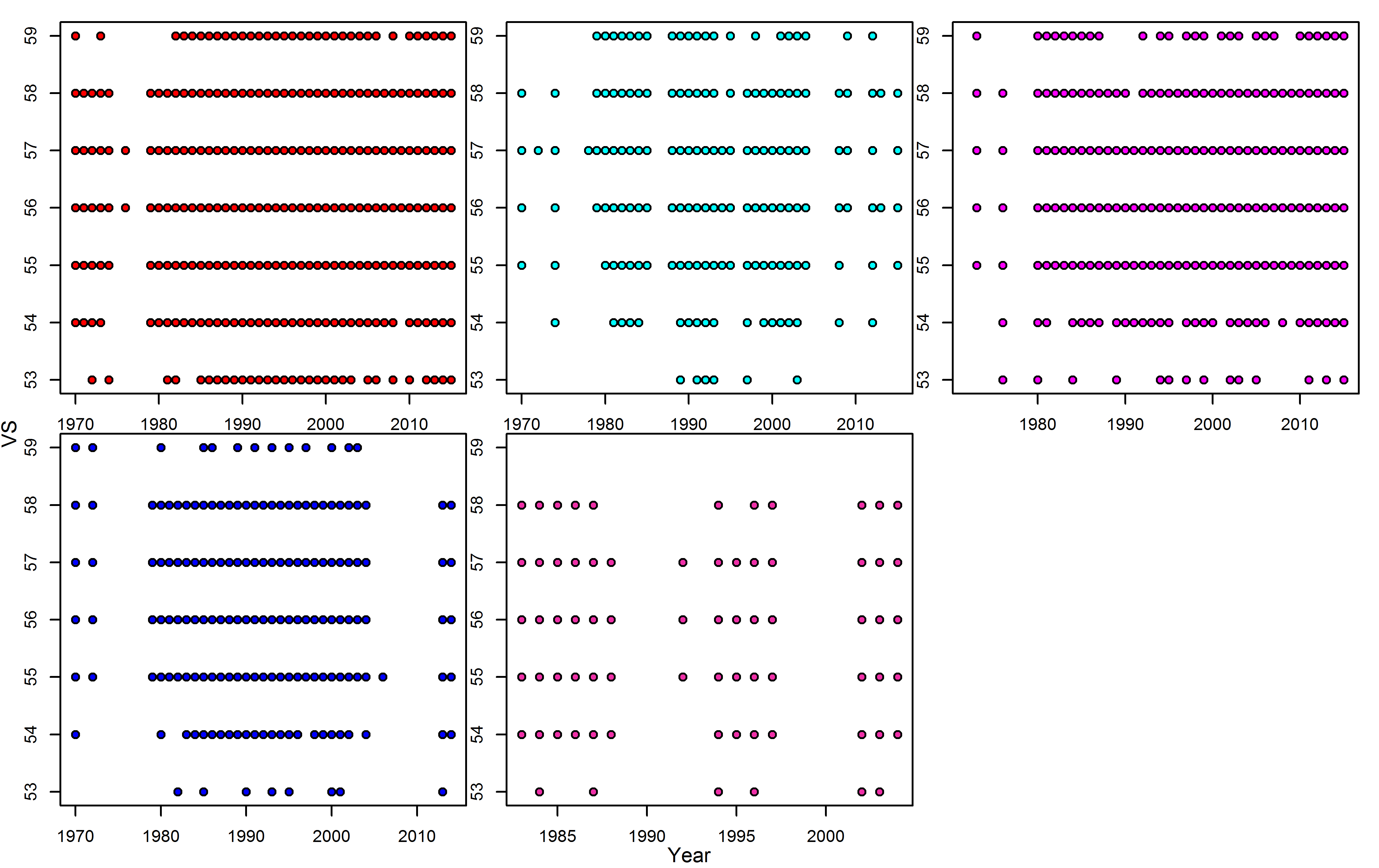

Supplement: S8 Fig — The low explained variance of only 17% for the GAM is resulting from the similar range and variance of vertebrae counts for the different areas (red = North Sea, cyan = west coast, purple = east coast, blue = Skagerrak, pink = western Baltic). (TIF) [file pone.0187374.s008.tif]

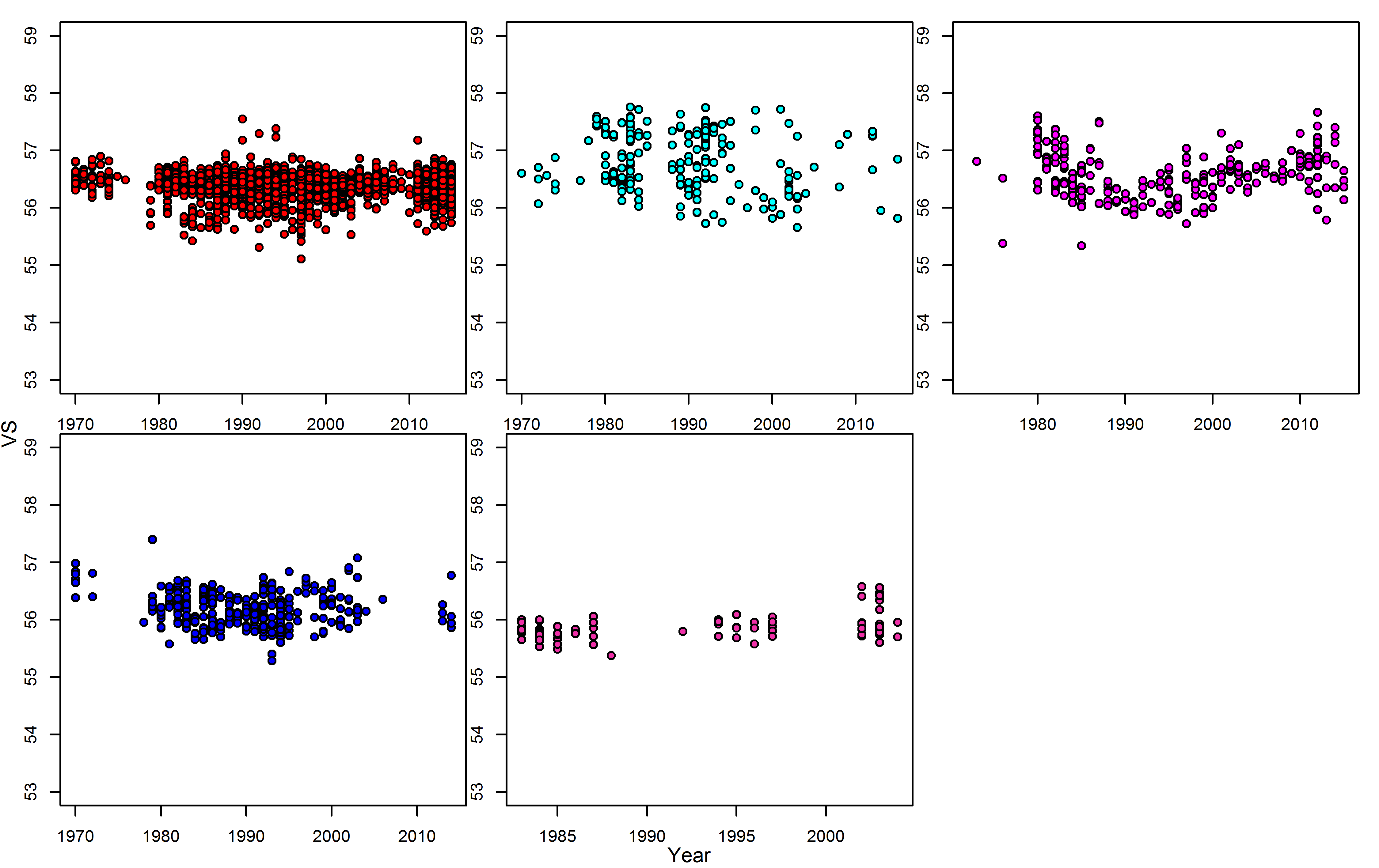

Supplement: S9 Fig — This data was not used in the generalized additive model (GAM) analysis, but would have increased the low explained variance of only 17% for the GAM, because the range and variance of vertebrae counts differs for the five areas (red = North Sea, cyan = west coast, purple = east coast, blue = Skagerrak, pink = western Baltic). (TIF) [file pone.0187374.s009.tif]
