# Supplementary material for: Comparative biology and population mixing among local, coastal and offshore Atlantic herring (Clupea harengus) in the North Sea, Skagerrak, Kattegat and western Baltic
Source: PLoS One. 2017 Oct 30;12(10):e0187374. doi: 10.1371/journal.pone.0187374 (PMC5662228; doi:10.1371/journal.pone.0187374)
Supplement: S1 Supporting Information — (PDF) [file pone.0187374.s010.pdf]

S1 Supporting information

Materials and methods

Study area

The study area was divided and all fish classified as: North Sea, west coast of Norway, east coast of Norway, Skagerrak, Kattegat, western Baltic, Nordfjord, Sognefjorden, Hardangerfjorden, Lysefjorden, Landvikvannet, Oslofjorden and Limfjorden based on their catch locations. The North Sea constitutes the area classified by ICES as the North Sea management area, bounded for this study in the south by 53.5°N. In addition, any fish occurring within 12 nautical miles (NM) of the Norwegian coastline were excluded from this area. Coastal areas were defined as areas within 12 NM off the Norwegian coast. The division between west and east was at Lista (7°E). ICES Division 3a includes the Skagerrak (Subdivision 20) and Kattegat (Subdivision 21); again with the exception of any fish occurring within 12 NM of the Norwegian coastline. The western Baltic is defined by the ICES Subdivision 22-24. Nordfjord, Sogne- and Lysefjorden are bounded in the west by 5.5°E and Hardangerfjorden by 5.8°E. Landvikvannet samples were taken within the brackish lake. Oslofjorden is bounded in the south by 59.4°N. Limfjorden samples were collected within 9.4-10.3°E.

Data analysis

Prior the generalized additive model (GAM) analysis, we applied a data exploration including the variables: Total length, age, number of vertebrae, stage of maturity, catch area, quarter of the year, temperature, salinity and fishing gear. During the data exploration, response and explanatory variables were check for outliers and for collinearity, and relationships between response and explanatory variables investigated. Only outliers for the number of vertebrae were removed for the model analysis. No correlation between any of the used variables were identified.

The following steps of model selection and validation were applied for the GAMs. After estimating the model, covariate with largest and non-significant *p*-value was removed. New models were estimated until all non-significant variables were removed. The final models were validated (see Fig S4-7) by (i) assessing normality (QQ-plot and histogram), (ii) homogeneity (residuals versus fitted values), (iii) model fit (fitted values versus observed values), and (iv) still existing patterns in relation to covariates (scatter plot of residuals vs. remaining covariates). None of these four validations of the final models were violated.
