# Supplementary material for: Comparative biology and population mixing among local, coastal and offshore Atlantic herring (Clupea harengus) in the North Sea, Skagerrak, Kattegat and western Baltic
Source: PLoS One. 2017 Oct 30;12(10):e0187374. doi: 10.1371/journal.pone.0187374 (PMC5662228; doi:10.1371/journal.pone.0187374)
Supplement: S1 Table — (PDF) [file pone.0187374.s011.pdf]

Table S1. Total number of analyzed herring per year per area 1970-2015.

Year	North Sea	West coast	East coast	Skagerrak	Kattegat	Western Baltic	Nordfjord	Sogne-fjorden	Hardanger-fjorden	Lyse-fjorden	Landvik-vannet	Oslo-fjorden	Lim-fjorden
1970	3 000	77		477				100					
1971	1 370			179									
1972	1 684	217	91	301									
1973	2 100	53	95	200									
1974	2 455	257											
1975	2 910	100		57									
1976	1 883		194				100						
1977	900	315	300	100				24	17				
1978	100	278	190	300				100					
1979	563	616		591	158			58	40	80			
1980	1 120	1 095	1 999	541	969			87	45	142			
1981	3 171	1 220	1 200	449	668								
1982	2 219	1 362	1 800	1 683									
1983	5 451	1 658	2 627	2 411		1 035							119
1984	9 570	1 086	2 200	1 282	155	2 829					134		295
1985	8 911	697	1 545	3 041	931	904	100		100	79	100		
1986	10 051	675	700	3 080	427	424						95	231
1987	12 241	500	624	875	469	691		100	300	196			166
1988	9 824	405	900	1 100	500	48	195	482	237	400			
1989	10 761	1 256	400	781	300		156	172		200			
1990	8 698	1 435	500	1 466	400		96	247	316	200		100	
1991	10 000	1 725	600	1 842	469		41	135	174	159	100		
1992	7 407	3 967	940	2 823	550	100	213	1146	139	52		243	
1993	8 330	2 056	200	2 595	575		100	28	100	213	100	92	
1994	6 542	547	509	2 484	100	320		200	201	395	56	200	
1995	7 842	856	1 071	985	193	459	99	100	149	200	100	596	70
1996	3 993	794	548	544	231	341	100	171	146	399	100	210	
1997	6 260	2 106	558	338		580	100	300	275	200	100	46	
1998	5 050	2 915	476	400			50	236	52	100	100	100	
1999	5 405	2 057	1 261	999	100		55	258	547	506	50	326	
2000	5 139	1 861	804	719	38		210	485	815	700	100	399	96
2001	5 608	434	686	783			200	322	575	292	100		
2002	9 133	785	1 231	1 456	200	1 242	459	356	621	438		687	100
2003	7 826	665	1 213	1 016	300	1 348	116	441	476	29		82	100
2004	4 494	111	430	100		199		146	310	114		145	
2005	4 465	230	441					857	144	397		131	
2006	6 846	103	401	100			512	24	38	100		157	
2007	4 269		322	208			140	366	213	160		180	
2008	3 218	161	575				710	671	187	102		390	
2009	3 340	1 490	146						508		100		
2010	4 369	1 211	650						371		246		
2011	7 183	1 138	524					142			261		
2012	11 525	720	1 231								377		
2013	11 750	773	324	200							96		
2014	7 214	11	393	382			87	156			45	100	
2015	11 883	1 057	542				100	245	18		261		
**Total**	**268 073**	**41 075**	**31 441**	**36 888**	**7 733**	**10 520**	**3 939**	**8 155**	**7 114**	**5 853**	**2 526**	**4 279**	**1 177**
