# Supplementary material for: Comparative biology and population mixing among local, coastal and offshore Atlantic herring (Clupea harengus) in the North Sea, Skagerrak, Kattegat and western Baltic
Source: PLoS One. 2017 Oct 30;12(10):e0187374. doi: 10.1371/journal.pone.0187374 (PMC5662228; doi:10.1371/journal.pone.0187374)
Supplement: S2 Table — (PDF) [file pone.0187374.s012.pdf]

S2 Table. Total number of analyzed herring per month per area 1970-2015.

Month	North Sea	West coast	East coast	Skagerrak	Kattegat	Western Baltic	Nord-fjord	Sogne-fjorden	Hardanger - fjorden	Lyse-fjorden	Landvik-vannet	Oslo-fjorden	Lim-fjorden
1	14 597	240	525	1 761	231	1 131							
2	20 847	4 719	2 989	174		611							
3	7 269	14 558	6 137	993	218	1 033					109		
4	4 729	4 141	3 812	913	100	2 497			362		344		753
5	23 594	2 826	1 203	1 690	848	952			126	109	1 826	43	424
6	57 388	2 031	896	4 501	100	424		10	100		237		
7	72 829	4 365	567	10 999	138	230		500	44				
8	9 820	1 597	724	7 017	458	586					10		
9	8 354	716	1 078	1 230		658				50		100	
10	15 856	2 085	1 855	500			87	246	150	200		100	
11	25 118	3 214	10 338	4 708	2 577	2 001	3 383	6 050	5 955	5 163		3 599	
12	7 672	583	1 317	2 402	3 063	397	469	1 349	377	331		437	
**Total**	**268 073**	**41 075**	**31 441**	**36 888**	**7 733**	**10 520**	**3 939**	**8 155**	**7 114**	**5 853**	**2 526**	**4 279**	**1 177**
