# Supplementary material for: Comparative biology and population mixing among local, coastal and offshore Atlantic herring (Clupea harengus) in the North Sea, Skagerrak, Kattegat and western Baltic
Source: PLoS One. 2017 Oct 30;12(10):e0187374. doi: 10.1371/journal.pone.0187374 (PMC5662228; doi:10.1371/journal.pone.0187374)
Supplement: S3 Table — (PDF) [file pone.0187374.s013.pdf]

S3 Table. Total number of analyzed herring by fishing gear per area 1970-2015.

Fishing gear	North Sea	West coast	East coast	Skagerrak	Kattegat	Western Baltic	Nord-fjord	Sogne-fjorden	Hardanger-fjorden	Lyse-fjorden	Landvik-vannet	Oslo-fjorden	Lim-fjorden
Trawl	117 475	12 939	10 088	8 893	6 750	100	3 852	7 823	6 870	5 694		4 179	
Seine	114 160	24 026	7 127	7 055			87	256	244	159		100	
Gillnet	469	3 788	14 105	497				76			2 526		
Hook and line	32	77		108									
Unspecific	35 937	245	121	20 335	983	10 420							1 177
**Total**	**268 073**	**41 075**	**31 441**	**36 888**	**7 733**	**10 520**	**3 939**	**8 155**	**7 114**	**5 853**	**2 526**	**4 279**	**1 177**
